# Simulation of Enhanced Growth of Marine Group II *Euryarchaeota* From the Deep Chlorophyll Maximum of the Western Pacific Ocean: Implication for Upwelling Impact on Microbial Functions in the Photic Zone

**DOI:** 10.3389/fmicb.2020.571199

**Published:** 2020-09-11

**Authors:** Jinlong Dai, Qi Ye, Ying Wu, Miao Zhang, Jing Zhang

**Affiliations:** State Key Laboratory of Estuarine and Coastal Research, East China Normal University, Shanghai, China

**Keywords:** mesoscale eddies, Marine Group I (MGI) *Thaumarchaeota*, Marine Group II (MGII) *Euryarchaeota*, deep chlorophyll maximum, simulation

## Abstract

Mesoscale eddies can have a strong impact on regional biogeochemistry and primary productivity. To investigate the effect of the upwelling of seawater by western Pacific eddies on the composition of the active planktonic marine archaeal community composition of the deep chlorophyll maximum (DCM) layer, mesoscale cold-core eddies were simulated *in situ* by mixing western Pacific DCM layer water with mesopelagic layer (400 m) water. Illumina sequencing of the 16S rRNA gene and 16S rRNA transcripts indicated that the specific heterotrophic Marine Group IIb (MGIIb) taxonomic group of the DCM layer was rapidly stimulated after receiving fresh substrate from 400 m water, which was dominated by uncultured autotrophic Marine Group I (MGI) archaea. Furthermore, niche differentiation of autotrophic ammonia-oxidizing archaea (MGI) was demonstrated by deep sequencing of 16S rRNA, *amoA*, and *accA* genes, respectively. Similar distribution patterns of active Marine Group III (MGIII) were observed in the DCM layer with or without vertical mixing, indicating that they are inclined to utilize the substrates already present in the DCM layer. These findings underscore the importance of mesoscale cyclonic eddies in stimulating microbial processes involved in the regional carbon cycle.

## Introduction

Mesoscale eddies are considered oceanic hotspots of prokaryotic activity. Microbial community structure varies greatly from surface waters (i.e., epipelagic zone) to deeper regions (e.g., mesopelagic zone) where different microbial community structures are found (Baltar et al., 2010). Previous studies have reported that mesoscale eddies play an essential role in controlling the distribution of microbial populations in the ocean (Ewart et al., 2008; Fong et al., 2008; Zhang et al., 2009, 2011; Liu et al., 2017). The deep chlorophyll maximum (DCM) is the layer of subsurface ocean water with the highest concentration of chlorophyll and thus high photosynthetic activity. Although depth can vary by season and location, the DCM is typically found between 60 and 120 m below the surface (Estrada et al., 1993). Generally, the DCM is located close to the bottom of the photic zone connecting to nutrient-rich deep waters (Martin-Cuadrado et al., 2015). The DCM provides phytoplankton and marine microbes with light and inorganic nutrients (Ghai et al., 2010). The distribution of Marine Group I (MGI) *Thaumarchaeota* (Fuhrman et al., 1992), Marine Group II (MGII) *Euryarchaeota* (DeLong, 2006; Ghai et al., 2010; Martin-Cuadrado et al., 2015), and Marine Group III (MGIII) *Euryarchaeota* (Galand et al., 2010; Haro-Moreno et al., 2017; Parada and Fuhrman, 2017) in the DCM zones have been characterized from different oceanic regions, however, interaction between these archaea group in the DCM and deeper waters is not well-studied.

MGII *Euryarchaeota* are globally distributed throughout the water column in the seas and open ocean (Massana et al., 2000). Based on 16S rRNA gene sequences, MGII has been classified into at least four groups: MGIIa, MGIIb, MGIIc, and MGIId (Belmar et al., 2011; Martin-Cuadrado et al., 2015), with MGIIb members notably abundant in surface waters and in the DCM (Massana et al., 2000). It was suggested that MGIIb should be grouped as a distinct class of euryarchaea, specifically *Thalassoarchaea* (Martin-Cuadrado et al., 2015). More recently, Rinke et al. (2019) proposed the new name *Candidatus* Poseidoniales, based on normalized ranks, comprising the families *Candidatus* Poseidoniaceae fam. nov. (formerly MGIIa) and *Candidatus* Thalassarchaeaceae fam. nov. (formerly MGIIb).

To date, there are no cultured isolates of MGII species. The ecological roles of MGII members have been revealed only by genome sequencing studies (Iverson et al., 2012; Martin-Cuadrado et al., 2015; Xie et al., 2018). In general, MGII members can degrade high-molecular-weight (HMW) organic matter such as proteins, carbohydrates, and lipids (Li et al., 2015). Amino acids, simple sugars, and fatty acids can serve as viable carbon sources for MGII growth (Martin-Cuadrado et al., 2015). The identification of proteorhodopsins in genomes derived from both surface and DCM layer indicated that MGII are a taxon of photoheterotrophs (Frigaard et al., 2006; Pereira et al., 2019). Based on the unique metabolic characteristics of the MGIIa and MGIIb genomes, 17 subclades have been identified, revealing different ecological patterns (Tully, 2019). These subclades include algal-saccharide-degrading coastal subclades, protein-degrading oligotrophic surface ocean subclades, and mesopelagic subclades lacking proteorhodopsins (Tully, 2019). These ecological distribution and metabolic profile of MGII species make them significant contributors to the global oceanic carbon cycle (Zhang et al., 2015).

In contrast to MGII *Euryarchaeota*, the availability of pure and enriched cultures of MGI *Thaumarchaeota* has led to the discovery that these organisms are chemolithotrophic ammonia oxidizers (Könneke et al., 2005; Santoro and Casciotti, 2011; Tourna et al., 2011; Qin et al., 2014; Jung et al., 2018). Although oceanic ammonia and nitrite oxidation are balanced, ammonia-oxidizing archaea (AOA) vastly outnumber the main nitrite oxidizers, the bacterial *Nitrospinae* (Kitzinger et al., 2020). The habitat specificity of AOA varies widely in terms of global phylogenetic (Alves et al., 2018). To date, all cultivated planktonic MGI representatives possess the homologs of the ammonia monooxygenase (AMO) subunit A (*amoA*) gene, which encodes a key enzyme catalyzing the oxidation of ammonia at extremely low concentrations (Santoro et al., 2019). To adapt to nutrient-limited conditions, marine thaumarchaeotal ammonia oxidizers commonly utilize a modified version of the 3-hydroxypropionate/4-hydroxybutyrate (3HP/4HB) cycle, which is the most energy-efficient aerobic pathway for CO_2_ fixation (Könneke et al., 2014). In the open ocean, the relative abundance of *Thaumarchaeota* sharply increases in the upper mesopelagic layer and decreases at depths greater than 1000 m (Karner et al., 2001). Evidences suggested that dark CO_2_ fixation by *Thaumarchaeota* reflects its high potential for primary production in mesopelagic waters (Ingalls et al., 2006; Reinthaler et al., 2006; Bergauer et al., 2013).

The tropical equatorial region of the Northwestern Pacific represents a typical oligotrophic marine environment with a euphotic zone generally less than 120 m deep (Zheng et al., 2015). Mesoscale eddies have been frequently observed in this region (Mizuno and White, 1983; Kawamura et al., 1986; Itoh and Yasuda, 2010). For example, the cyclonic Mindanao Eddy (ME) exists year-round center at 7°N, 128–130°E, and impacting water layers from 50 to 500 m (Zhang et al., 2012). Several studies have examined the physical processes associated with mesoscale eddies (White and Annis, 2003; Itoh and Yasuda, 2010; Kouketsu et al., 2011). Mesoscale eddies as well as other diapycnal mixing processes provide an important mechanism for the exchange of nutrients, dissolved gases, and particulate matter between the shallow and deep layers (Tian et al., 2009). Under the influence of the cold eddies, new nutrients and organic matter are brought to the DCM. This results in changes in microbial heterotrophic activity of the water layer relative to the surrounding area (Ewart et al., 2008). Systematic differences in bacterial responses within and between cyclonic and mode-water eddies have been documented in the Sargasso Sea (Ewart et al., 2008). Bacterial community structures in two cold-core cyclonic eddies in the South China Sea were significantly influenced by cyclonic eddy perturbations, causing a shift in the microbial community in the euphotic zone (Zhang et al., 2011). Robidart et al. (2018) conducted a mixed experiment in the subtropical circulation of the North Pacific and revealed that deep-water nutrients and particles had distinct effects on key members of the surface community, including *Prochlorococcus*, *Synechococcus*, eukaryotic phytoplankton, N_2_-fixing cyanobacteria, and viruses. The contribution of MGI *Thaumarchaeota* to total picoplankton abundance and total active cells increased with depth, and the contribution of MGI in the upper mesopelagic water was greater inside the cyclonic eddy system relative to outside the system in the South China Sea (Zhang et al., 2009). However, little is known about how the western Pacific mesoscale eddies influence the active archaeal community (especially MGII) in the DCM.

Here, we conducted a set of small-scale mixing experiments to better characterize the effect of mesoscale eddies on the relationships in archaea communities between DCM and 400 m mesopelagic waters. Specifically, we studied shifts in archaeal communities of the DCM that occurred in response to vertical mixing with 400 m water through Illumina sequencing of the 16S rRNA gene and 16S rRNA transcripts. We hypothesized that heterotrophic microorganisms in the DCM layer could grow rapidly after being stimulated by fresh substrates, which were brought from the 400 m waters and produced by autotrophic microbes, thereby promoting the metabolism of organic matter in DCM layer and affecting the regional carbon cycle.

## Materials and Methods

### *In situ* Seawater Sample Collection

**FIGURE 1:**
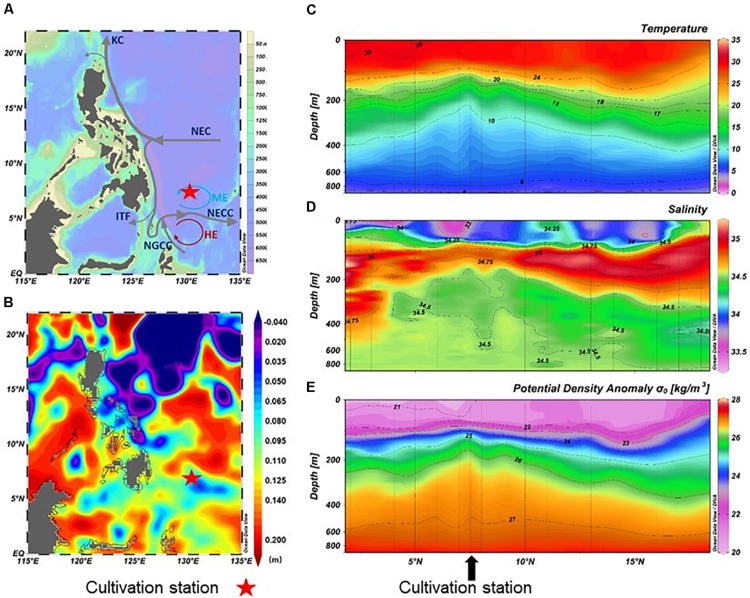
Map showing the sampling station **(A)**, sea surface height **(B)**, and distribution of temperature **(C)**, salinity **(D)**, and potential density anomaly σt **(E)** in the upper 1000 m. KC, Kuroshio Current; NEC, North Equatorial Current; NECC, North Equatorial Countercurrent; ITF, Indonesian throughflow; NGCC, New Guinea Coastal Current; ME, Mindanao Eddy; HE, Halmahera Eddy. Dataset of sea surface height was downloaded from Archiving Validation and Interpolation of Satellite Oceanographic Data, AVISO; http://www.aviso.altimetry.fr/

A culture experiment was conducted during the R/V *Kexue* cruise (October 15th, 2017–November 16th, 2017) in the western Pacific Ocean (Figure 1). DCM (90 m) and mesopelagic (400 m) seawater samples were collected and stored in sterile barrels at 7.75° N, 130° E on October 31, 2017.

### Vertical Mixing Culture Experiment

**FIGURE 2:**
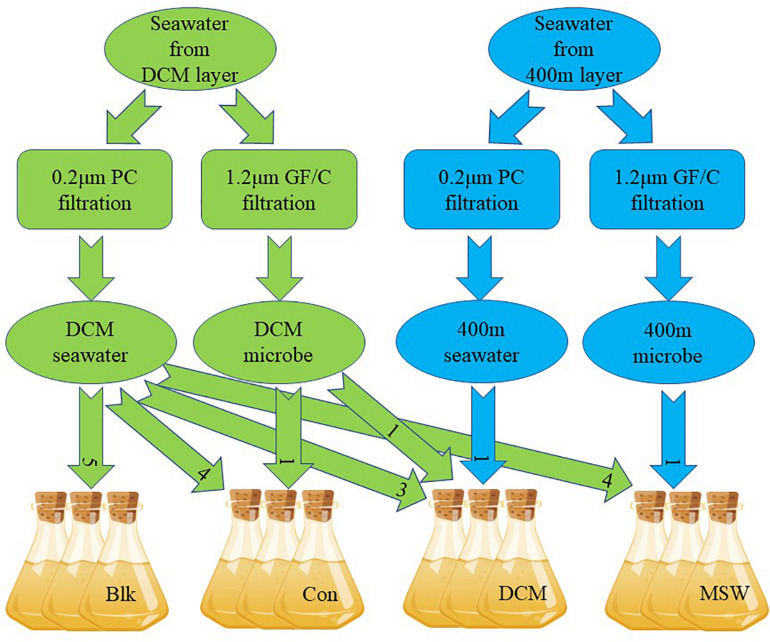
Simulation of the water mixing cultivation experiment. DCM seawater means DCM seawater filtered through a 0.2 μm polycarbonate filter, DCM microbe means DCM seawater filtered through a 1.2 μm glass microfiber filter, 400 m seawater means 400 m seawater filtered through a 0.2 μm polycarbonate filter, 400 m microbe means 400 m seawater filtered through a 1.2 μm glass microfiber filter. Each culture system was 4.5 L in volume, and three parallel experiments were conducted. Seawater and microbes were always mixed in a 4:1 ratio. Blk was checked for the removal of microorganisms from seawater. The number in the arrow represents the mixing ratio. All experiments were performed at 18°C in the dark for 24 h.

The mixed culture experiment in this study simulated mixing of deep seawater (400 m) and the surface DCM layer (90 m) by mesoscale cyclonic cold eddies in the western Pacific. Compared with the DCM layer, the 400 m layer is a high-pressure, low-temperature marine environment with little sunlight. Sixty liters of DCM seawater and 10 L of 400 m seawater were collected on October 31, 2017 (Figure 1). Among these samples, 45 L of DCM seawater and 3 L of 400 m seawater were filtered with a 0.22 μm Nuclepore polycarbonate membrane filter (Whatman, NJ, United States) to remove microorganisms, yielding the “DCM ultrafiltrate.” Next, 10 L of DCM seawater and 3 L of 400 m seawater were filtered with a 1.2 μm GF/C membrane (Whatman, NJ, United States) to remove phytoplankton, yielding the “DCM microbes” or “400 m microbes”, respectively. All filtered water samples were kept in dark, sterile barrels before mixing. A total of four groups were set up in this microcosm experiment, with three parallel in each group: (1) only DCM ultrafiltrate (= Blk); (2) DCM ultrafiltrate + DCM microbes at a 4:1 v/v ratio (= Con); (3) DCM ultrafiltrate + DCM microbes + 400 m ultrafiltrate in a 3:1:1 v/v ratio (= DCM); and (4) DCM ultrafiltrate + 400 microbes in a 4:1 v/v ratio (= MSW). The volume of each parallel after mixing was 4.5 L (Figure 2). The mixed samples were all placed in a temperature-controlled laboratory at 18°C for 24 h and shaken once in the middle of the incubation to ensure that samples remained evenly mixed.

All the samples were filtered through 0.22 μm Nuclepore polycarbonate membrane filters (Whatman, NJ, United States) at the end of the incubation experiment. These membrane samples were stored in liquid nitrogen until RNA/DNA extraction. Approximately 2 L of each sample was filtered through 0.22 μm Nuclepore polycarbonate membrane filters (Whatman, NJ, United States) at the end of the incubation experiment. These membrane samples were stored in liquid nitrogen until RNA/DNA extraction. Because of the limitations associated with the field culture experiment conditions, we could only use one of the three parallel groups for RNA extraction, and the other two are used for DNA extraction for 16S rRNA, *amoA*, and *accA* gene detection, while monitoring the parallel experimental groups. Dissolved organic carbon (DOC) samples were filtered through 0.4 μm nylon filters (Rephile, Shanghai, China), and nutrient samples were filtered through 0.4 μm Nuclepore polycarbonate membrane filters (Whatman, NJ, United States); samples were then stored in a freezer at −20°C.

### Chemical Parameter Analysis

After the DOC samples were acidified, oxygen was purged to remove dissolved inorganic carbon, and samples were analyzed with a total organic carbon (TOC) analyzer (TOC L-CPH, Shimadzu, Japan) using the high-temperature catalytic oxidation method. Nutrients (silicate, phosphate, and nitrite) were determined photometrically by an auto-analyzer (Model: Skalar SAN^plus^) with precision of < 5–10%. Seawater references were run with each batch of samples to check the precision of nutrient analysis.

### DNA and RNA Extractions

Total DNA was extracted from two randomly selected replicate filters from the 24 h (T24) samples using a MoBio PowerSoil^®^ DNA Isolation Kit (MOBIO Laboratories, Carlsbad, CA, United States). Total RNA was extracted from the last filter from the 24 h (T24) samples using an E.Z.N.A. Soil RNA Mini Kit (Omega Bio-Tek, Norcross, GA, United States). The RNA was subsequently converted to cDNA using the HiScript^TM^ Q RT SuperMix for qPCR (+ gDNA wiper) Kit (Vazyme Biotech, China). Final concentrations and purity of the DNA and cDNA were measured spectrophotometrically with a NanoDrop ND2000 (Thermo Fisher Scientific, Wilmington, DE, United States). The extracted DNA and cDNA were stored at −80°C until further analysis.

### PCR Amplification, Illumina MiSeq Sequencing, and Sequence Data Processing

To decrease polymerase chain reaction (PCR) bias, we performed the minimum number of PCR cycles required to produce a product; furthermore, three independent PCR mixtures were pooled for each sample. The V4–V5 hypervariable regions of the archaeal 16S rRNA genes were amplified using the primers 524F10extF (5′-TGYCAGCCGCCGCGGTAA-3′) and Arch958RmodR (5′-YCCGGCGTTGAVTCCAATT-3′) (Pires et al., 2012) and the following amplification conditions: denaturation at 95°C for 3 min; 35 cycles of denaturation at 95°C for 30 s, annealing at 55°C for 30 s, and extension at 72°C for 45 s; and an extension at 72°C for 10 min and cooling at 4°C. The V4–V5 hypervariable regions of the bacterial 16S rRNA gene were amplified using the primers 515F (5′-GTGCCAGCMGCCGCGG-3′) and 907R (5′-CCGTCAATTCMTTTRAGTTT-3′) (Xiong et al., 2012) and the following amplification conditions: denaturation at 95°C for 2 min; 25 cycles of denaturation at 95°C for 30 s, annealing at 55°C for 30 s, and extension at 72°C for 30 s; and an extension at 72°C for 10 min and cooling at 4°C. The Archaeal *amo*A gene fragments were amplified using the primers Arch-*amoA*F and Arch-*amoA*R (5′-GCGGCCATCCATCTGTATGT-3′) (Francis et al., 2005) and the following amplification conditions: denaturation at 95°C for 3 min; 35 cycles of denaturation at 95°C for 30 s, annealing at 55°C for 30 s, and extension at 72°C for 45 s; and an extension at 72°C for 10 min and cooling at 4°C. The *acc*A gene fragments were amplified using primers Cren529F (5′-GCWATGACWGAYTTTGTYRTAATG-3′) and Cren981R (5′-TGGWTKRYTTGCAAYTATWCC-3′) (Yakimov et al., 2009) and the following amplification conditions: denaturation at 95°C for 4 min; 35 cycles of denaturation at 95°C for 40 s, annealing at 51°C for 40 s, and extension at 72°C for 90 s; and an extension at 72°C for 10 min and cooling at 4°C.

For Illumina MiSeq sequencing, PCR products were purified using the AxyPrep DNA Gel Extraction Kit (Axygen Biosciences, United States) per the manufacturer’s protocol and then quantified by QuantiFluorTM-ST (Promega, United States). Reaction mixtures were pooled in equimolar ratios and paired-end reads were generated on an Illumina MiSeq PE250 (Majorbio Bio-Pharm Technology Co., Ltd., Shanghai, China).

### Sequence Data Processing, OTU Clustering, and Taxonomic Assignment

Raw Illumina FASTQ files were demultiplexed, quality-filtered, and analyzed using Quantitative Insights into Microbial Ecology (QIIME) (version 1.17) (Caporaso et al., 2010) using criteria described previously (Li et al., 2014). Operational taxonomic units (OTUs, 97% similarity cutoff) were clustered using UPARSE (version 7.1)^1^. Chimeric sequences were screened using UCHIME. The abundances of OTUs from each sample were determined by OTU clustering. Reads from each sample were assigned to each OTU, and an OTU table was generated using the “usearch_global” command. To obtain the taxonomic information for each species corresponding to an OTU, the Ribosomal Database Project (RDP) Classifier^2^ was used for taxonomic analysis of representative OTU sequences. The community composition of each sample was calculated at the genus level.

### Phylogenetic Analyses

The sequences of the representative OTUs in this study were blasted against GenBank by BLAST^3^ to obtain reference sequences. The sequences of representative OTUs and selected reference sequences from the database were aligned using Clustal W. A maximum likelihood phylogenetic tree was generated in MEGA6 using the neighbor-joining method with 1000 bootstrap replicates (Tamura et al., 2013) and was visualized in iTOL (Letunic and Bork, 2007).

### Statistical Analyses

Alpha diversity metrics, principal coordinates analyses (PCoAs) using Bray–Curtis distances, and inter-group differential species test based on chi-square test were performed using the free online Majorbio I-Sanger Cloud Platform^4^.

### Nucleotide Sequence Accession Numbers

Sequence data in this study were entered into the NCBI Sequence Read Archive (SRA) under BioProject ID PRJNA511510 and PRJNA634930.

## Results

### Description of Site Environmental Characteristics

The sampling collection station was located close to a mesoscale eddy center (ME, 7°N, 128–130°E). *In situ* physical profiles (i.e., temperature and salinity as a function of depth) were obtained from CTD information. In the area affected by ME at 130°E, the sea level was lower than the surrounding areas, and the temperature, salinity, and density lines were markedly increased (Figure 1 and Supplementary Table S1). At 90 m, the temperature was 24.0°C, salinity was 34.8, oxygen concentration was 5.2 mg/L, and the concentration of DOC was 59 μmol/L. In contrast, the region at 400 m was a low-temperature and high-pressure environment. The temperature dropped to 7.7°C, the salinity changed slightly (34.5‰), and the oxygen and DOC concentration decreased to 3.1 mg/L and 47 μmol/L, respectively. In addition, the concentrations of nutrient (nitrate, silicate, and phosphate) in 400 m seawater was higher than that in 90 m seawater (Supplementary Table S1).

### Varies in Chemical Parameters During Cultivation

**FIGURE 3:**
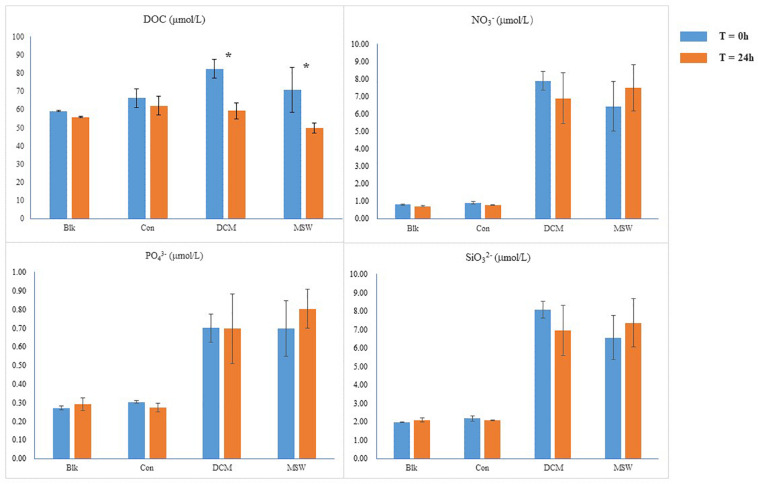
Chemical parameters in each group; DOC, dissolved organic carbon; * indicates a significant difference.

Since the DCM and MSW groups consisted of seawater from the 400 m layer, initial nutrient concentrations were higher than those in the Con group. After 24 h incubation experiments, the concentration of DOC decreased in the three groups. Compared with Con, DOC in DCM and MSW decreased significantly, especially in the DCM group (by 23 μmol/L). Mixing resulted in higher initial nitrate content in the DCM combined MSW group than those in the other three groups, and after 24 h, nitrate was consumed in the DCM group and accumulated in the MSW group. In addition, silicate and phosphate levels remained relatively stable throughout the experiment (Figure 3).

### Distribution and Composition of Total (DNA) and Active (RNA) Archaeal Communities in Each Mixing Group

**FIGURE 4:**
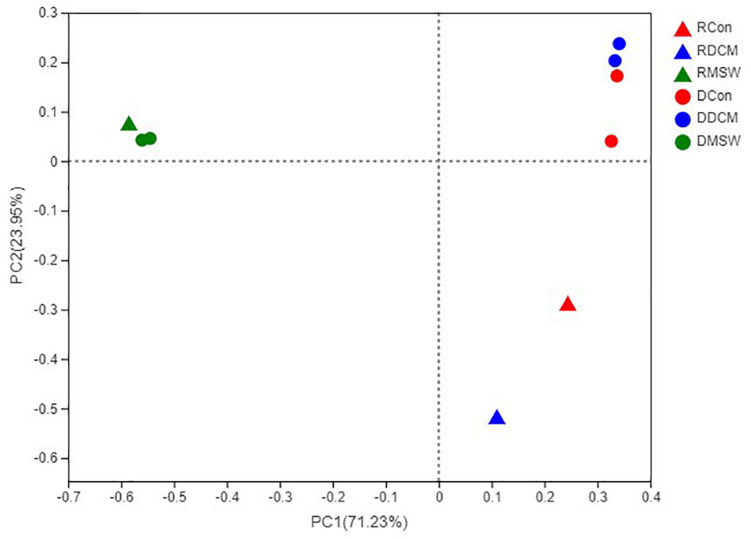
Principal coordinates analysis (PCoA) based on a Bray–Curtis distance matrix representing differences in the community structure of six groups at the OTU level. The first and second principal components (PCo1 and PCo2) are shown, explained 71.23 and 23.95% of the variance in the dataset, respectively. Triangles and circles indicate control active (RNA) and total (DNA) archaeal communities, respectively. Con, DCM, and MSW groups are represented by red, blue and green, respectively.

PCR was successfully performed with DCM, MSW, and Con samples, but no PCR products were obtained from Blank (Blk) samples (Supplementary Table S2). PCoA analysis based on the sequencing data showed consistent clustering of DNA samples in each group (Figure 4), indicating that the culture-based experiments were highly replicable. There were significant differences (*p* < 0.01) between RNA and DNA in both the Con and DCM groups, whereas the total and active communities were clustered in the MSW group. Our results revealed possible archaeal community in the DCM layer with and without vertical mixing. Furthermore, the sequencing results of the functional genes on the PCoA distribution distinguished the three groups clearly, and 16S rRNA, *amoA*, and *acc*A showed similar distribution trends. Specifically, the compositions of MSW in 16S rRNA, *amo*A, and *accA* were significantly different from those in the other two groups (*p* < 0.01) (Supplementary Figure S1).

After sequencing, we obtained a total of 413,804 high-quality archaeal V4–V5 Illumina sequences from both total (DNA) and active (RNA) communities. There were 31,051 archaeal reads per sample after subsampling. Based on the 97% similarity cutoff, there were 94 archaeal OTUs in the complete OTU dataset, and *Euryarchaeota* (20.3%) and *Thaumarchaeota* (78.9%) were the main phyla detected. The main families within *Euryarchaeota* were MGII and MGIII, and the most abundant class within *Thaumarchaeota* was MGI.

**FIGURE 5:**
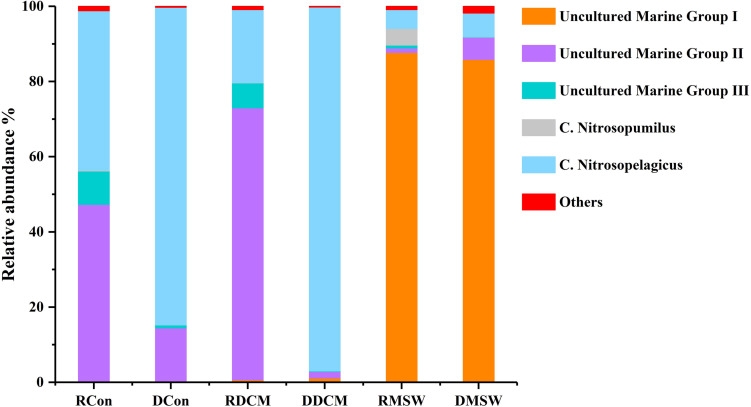
Distributions of genus-level taxa. Bars represent the relative abundance of Illumina sequences representative of each genus. Bacterial taxa represented by less than 1% of reads are pooled as “others.” “Uncultured” means that the specific archaeal taxa cannot be classified at the genus level. The R before the sample group name represents RNA (active), and D stands for DNA (total). The proportion of each sequence in DNA samples is the mean value of duplicate analyses.

Figure 5 shows that there were some variations in the percentage compositions among the groups at the genus level. For total archaeal communities, *Nitrosopelagicus* was the dominant genus in the Con (87.0%) and DCM (97.1%) samples, and uncultured MGII members made up 12.2 and 1.6% of the archaeal sequences in the Con and DCM groups, respectively. However, uncultured MGIII members and *Nitrosopumilus* were almost absent from these two groups (<1%). In the MSW group, uncultured MGI (87.6%) members was the most abundant genus; *Nitrosopelagicus* (6.4%) and uncultured MGII members (5.9%) were two other abundant genera; and *Nitrosopumilus* and uncultured MGIII members were less abundant than these three genera (<1%). In contrast to the total archaeal communities, the active MGII archaea represented 46.6 and 72.5% of reads in the Con and DCM samples, respectively, and active MGIII made up 9.2% (Con sample) and 6.8% (MSW sample) of the sequences in total archaeal community. Uncultured MGI (86.8%) members were the most abundant archaea in the MSW group, demonstrating that the active archaeal communities of the MSW group were quite different from those of the DCM or Con groups.

### Distribution of MGI in Each Group

**FIGURE 6:**
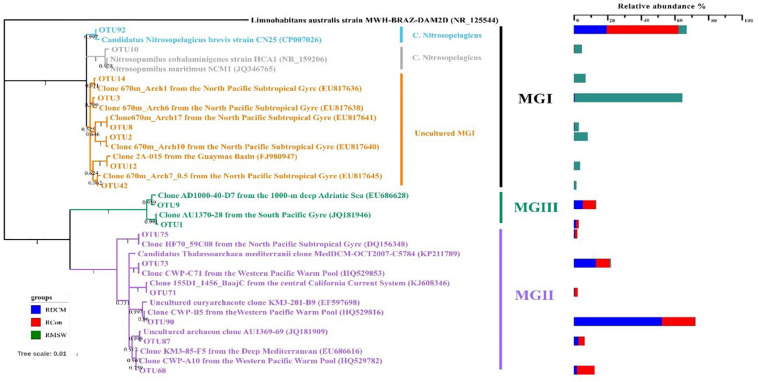
Neighbor-joining phylogenetic tree based on 16S rRNA gene sequences of the representative OTUs based on 16S rRNA gene sequences from NCBI GenBank. These OTUs were those that made up more than 1% of the reads from at least a single sample. The scale bar represents the estimated number of nucleotide changes per sequence position. Percentage on nodes refer to the percentage of recovery from 1000 bootstrap resamplings. Only values > 50% are shown. *Limnohabitans australis* strain MWH-BRAZ-DAM2D (NR_125544) was used as the outgroup. The color bar represents the percentage composition of reads in an RNA sample.

Eight MGI OTUs possessed more than 1% of the reads from either a single sample or from multiple samples. These OTUs formed three distinctive clusters (Figure 6). OTU92 in MGI cluster 1 had 99.5% similarity with *Candidatus* Nitrosopelagicus brevis strain CN25 (CP007026), an ammonia-oxidizing enrichment culture collected from a depth of 25m in the northeastern Pacific (35.46°N, 124.91°W) (Jung et al., 2018). This OTU was predominant in both DCM and Con samples at the DNA level, and the relative abundance of OTU92 reached up to 42.39% in the Con-RNA sample, whereas OTU92 accounted for 19.42% of the total DCM-RNA at the end of the culturing period. OTU10 in MGI cluster 2 included 4.45% of the total sequences from the MSW-RNA sample and displayed 99.1% similarity with *Nitrosopumilus cobalaminigenes* strain HCA1 (NR_159206) (Qin et al., 2017). The five OTUs in MGI cluster 3 were closely related to MGI clones from an archaeal community at 670 m in the mesopelagic zone of the North Pacific Subtropical Gyre (Hansman et al., 2009), and the sequences of OTU2, OTU3, OTU8, OTU14, and OTU42 possessed > 99.3% similarity with the corresponding sequences in these clones. Sequences of these five OTUs contributed the most to either active (83.11% of total sequences) or total (79.36% of total sequences) communities in 400-m deep water but were seldom recovered from all of the tested DCM and Con samples (Table 1).

**TABLE 1:** Relative abundance (%) of 16 OTUs represented by more than 1% of the reads from at least a single sample and their closest matches retrieved from NCBI GenBank.

**Phylogenetic group**				**Relative abundance (%)**						**Closest matches to 16S rRNA genes in NCBI database**	
		
				**Con**		**DCM**		**MSW**			
**Class**	**Family**	**Genus**	**OTU**	**RNA**	**DNA**	**RNA**	**DNA**	**RNA**	**DNA**	**Clone or culture (accession no.)**	**Similarity**
Thermoplasmata	MGII		OTU90	19.55	5.08	52.14	1.05	0.32	1.14	CWP-B5 (HQ529815)	100%
			OTU73	8.17	1.95	12.98	0.39	0.84	0.75	CWP-C7 (HQ529853)	100%
			OTU87	3.65	1.06	2.69	0.06	0	0.29	AU1369-69 (JQ181909)	99.8%
			OTU68	10.38	2.54	1.84	0.05	0.03	3.30	CWP-A10 (HQ529782)	100%
			OTU75	1.42	1.13	0.62	0.04	0	0.08	HF70_59C08 (DQ156348)	100%
			OTU71	1.85	0.07	0.35	0.00	0	0.03	155D1_1456_BaajC (KJ608346)	100%
	MGIII		OTU9	7.47	0.61	5.31	0.16	0.53	0.05	AD1000-40-D7 (EU686628)	99.3%
			OTU1	1.44	0.18	1.32	0.02	0.24	0.01	AU1370-28 (JQ181946)	99.8%
MGI		Uncultured MGI	OTU3	0.02	0.01	0.46	0.89	63.68	57.77	670m_Arch6 (EU817638)	99.3%
			OTU2	0	0	0	0	8.24	8.11	670m_Arch10 (EU817640)	99.8%
			OTU14	0	0	0.04	0.08	6.90	9.63	670m_Arch1 (EU817636)	99.8%
			OTU12	0	0	0.01	0	3.72	3.46	2A-015 (FJ980947)	100%
			OTU8	0	0	0.16	0.14	2.76	1.47	670m_Arch17 (EU817641)	100%
			OTU42	0	0	0	0	1.53	2.38	670m_Arch7_0.5 (EU817645)	99.5%
		Nitrosopumilus	OTU10	0.22	0.00	0.07	0	4.45	0.06	Nitrosopumilus cobalaminigenes strain HCA1 (NR_159206)	99.1%
		*Ca.* Nitrosopelagicus	OTU92	42.39	84.44	19.42	96.72	4.99	6.07	*Candidatus* Nitrosopelagicus brevis strain CN25 (CP007026)	99.5%

*Two DNA parallel averages were used to calculate the relative abundance values in the table.*

### Diversity of MGII in Each Group

Six MGII OTUs were abundant (>1% and present in at least one sample) (Table 1). Among these OTUs, OTU90 had the highest relative abundance in the DCM-RNA sample (up to 52.14%), which was approximately three times higher than that in the Con-RNA sample (19.55%). This OTU represented only 0.32% of the total sequence abundance in the MSW-RNA sample. OTU90 was phylogenetically associated (100% similarity) with the clone CWP-B5 from the Ontong Java Plateau from the center of the western Pacific warm pool (HQ529815) and the clone KM3-85-F5 (99.5% similarity) from the deep Mediterranean, which was clustered in subcluster O of MGIIb (Galand et al., 2010). OTU90 had a 96% similarity with the metagenomic fosmid clone MedDCM-OCT2007-C57 from the Mediterranean DCM, which is named *Candidatus* Thalassoarchaea mediterranei (KP211789) and belongs to the class *Thalassoarchaea*. In addition, three other OTUs (OTU68, OTU73, and OTU87) had 96.8–98.2% similarity with *Ca.* Thalassoarchaea mediterranii (Figure 6).

### Diversity of Archaeal *amoA* and *accA* Genes

We analyzed seven representative OTUs of the AOA *amoA* gene (> 1% and appeared in at least one sample). These OTUs fell into two distinct phylogenetic clusters (Supplementary Figure S2). Four OTUs (OTU77, 78, 47, and 82) within the WCA cluster dominated in the DCM group (Table 2 and Supplementary Figure S3). OTU77 had a 99.3% similarity with *Ca.* Nitrosopelagicus brevis strain CN25 (CP007026), an ammonia-oxidizing enrichment culture collected from a depth of in the northeastern Pacific (35.46°N, 124.91°W). The relative abundances of this OTU reached 85.23% in Con and 85.04% in DCM, but only 12.08% in MSW. In contrast, three OTUs (OTU48, 54, and 63) within the WCB cluster dominated in the MSW group, but they are almost absent in Con and DCM (Table 2 and Supplementary Figure S3). Three representative OTUs retrieved from MSW were identical to clones in the same depth (400 m) from different oceans. For example, OTU54 had 100% similar with clone 712-400-amoA22 (GU181561) in the 400 m layer from the East China sea, and the other two OTUs (OTU48 and OTU63) had 100% similarity with clone 6-400m_07 (KC596418) and clone a109.400.46d (JF272642) in the 400 m water from the North Pacific and the Arctic Baffin Bay, respectively.

**TABLE 2:** Relative abundance (%) of seven *amoA* OTUs represented by more than 1% of the reads from at least a single sample and their closest matches retrieved from ammonia-oxidizing archaea (AOA) *amoA* genes in the Fungene and NCBI databases.

**OTU**	**Relative abundance (%)**			**Closest matches to AOA amoA genes in Fungene and NCBI database**	
		
	**Con**	**DCM**	**MSW**	**Clone or culture (accession no.)**	**Similarity**
OTU77	85.23	84.04	12.08	*Candidatus* Nitrosopelagicus brevis strain CN25 (CP007026)	99.3%
OTU78	4.41	5.04	1.38	*Candidatus* Nitrosopelagicus brevis strain CN25 (CP007026)	98.6%
OTU47	4.7	3.41	0.43	608-75-amoA29 (GU181598)	100%
OTU82	3.02	3.29	0.34	MW_AamoA_100m_06 (AB703976)	100%
OTU54	0	0.65	15.16	712-400-amoA22 (GU181561)	100%
OTU48	0	0	13.08	6-400m_07 (KC596418)	100%
OTU63	0	0	4.72	a109.400.46d (JF272642)	100%

*Two parallel averages were used to calculate the relative abundance values in the table.*

Based on the top three abundant OTUs of the *accA* gene in at least one sample, the phylogenetic tree showed two distinct clusters – one with sequences from Con and DCM and the other with sequences from MSW (Supplementary Figure S3) – a topology consistent with that for 16S rRNA and *amoA* genes. In the DCM group, OTU11 had the highest relative abundance in Con (59.29%) and DCM (53.40%) but was seldom detected in MSW (0.65%) (Table 3). OTU11 had 99.8% similar with the uncultured thaumarchaeote clone S100 accA 21 (GQ507517) found at a depth of 100 m from the South China Sea. The second most abundant OTU71 had 98.8% similarity with an uncultured thaumarchaeote clone (MF137423) from the East China Sea. In the MSW group, OTU21 showed 99.3% similarity with the uncultured thaumarchaeote clone D5-450m-accA-61 collected at a 450-m depth in the Gulf of Mexico, which was the most abundant in MSW (64.77%). Approximately 5.52% sequences in the DCM sample belonged to OTU21, but this OTU was not detected in the Con group (Table 3). Compared with OTU21, OTU15, and OTU55 had lower relative abundance but similar distribution trend (Table 3). Additionally, no closest match of OTU86 from the DCM layer was detected in the NCBI database, indicating that OTU86 may represent a novel species.

**TABLE 3:** Relative abundance (%) of 12 *accA* OTUs represented by the top three abundant reads from at least a single sample and their closest matches retrieved from NCBI GenBank.

**OTU**	**Relative abundance (%)**			**Closest matches to *accA* genes in NCBI database**	
		
	**Con**	**DCM**	**MSW**	**Clone or culture (accession no.)**	**Similarity**
OTU11	59.29	53.40	0.65	S100 accA 21 (GQ507517)	99.8%
OTU71	30.52	22.55	0.12	2014SepS39093 (MF137423)	98.8%
OTU21	0	5.52	64.77	D5-450m-accA-61 (KC349506)	99.3%
OTU15	0	0.36	7.66	Z500 accA 31 (GQ507619)	97.0%
OTU55	0	0.19	5.11	2014SepS6800118 (MF137701)	99.6%
OTU86	2.15	2.98	0	No match	

*Two parallel averages were used to calculate the relative abundance values in the table.*

### Distribution of Total (DNA) Bacterial Community in Each Mixing Group

After subsampling, we obtained 51,569 bacterial reads per sample in the total (DNA) community. Based on the 97% similarity cutoff, there were 107 OTUs in the complete OTU dataset. *Alphaproteobacteria* was the dominant class in all samples, ranging from 64.5 to 78.3% of the sequences. *Gammaproteobacteria* was the second most abundant class in all samples (15.9–24.4%). Class *Flavobacteriia* constituted 2.3–10.6% of the bacterial sequences. Less than 50 of the 51,569 sequences of the phylum *Cyanobacteria* were recovered in each group. Furthermore, no reads affiliated with known marine NOB, including phylum *Nitrospinae* and *Nitrospirae*, were detected in both datasets (Supplementary Figure S4).

## Discussion

### Niche Differentiation of MGI Between the DCM Layer and the 400 m Deep Water

In the open ocean, depth is a significant factor controlling the distribution of AOA (Francis et al., 2005; Mincer et al., 2007; Smith et al., 2016; Santoro et al., 2017). Phylogenetic analysis of thaumarchaeal *amo*A divided the marine *Thaumarchaeota* into two main clades based on depth: the shallow, water column “A” (WCA) (Francis et al., 2005; Hallam et al., 2006) ecotype and the deep, water column “B” (WCB) ecotype (Smith et al., 2016). The WCA clade is generally detected at all depths, with the peak abundance of the WCA *amo*A gene located near the top of the nitracline (Santoro et al., 2017), whereas the WCB clade was detected primarily below the photic zone (Beman et al., 2008; Santoro et al., 2010). *Ca.* Nitrosopelagicus brevis strain CN25 was the only cultivated representative of the shallow, WCA clade. There are currently no enrichments or isolates of the deep, WCB clade (Santoro et al., 2019). In our study, sequences related to the *Ca.* Nitrosopelagicus brevis strain CN25 were abundant in the total and active DCM archaeal communities, indicating that the WCA clade inhabited the DCM layer. Sequences of the six MGI OTUs belonging to the distinct uncultured MGI cluster were abundant in both active and total archaeal communities in the 400-m deep water sample. Closest relatives of these OTUs have been collected from 670 m of the North Pacific Subtropical Gyre (Hansman et al., 2009). These uncultured MGI archaeal groups were reported that adapted to thrive in mesopelagic waters and might possess an inorganic carbon fixation pathway (Hansman et al., 2009). Our data on the niche differentiation of MGI are consistent with previously observed ecotype-specific AOA in the water column of the Pacific (Smith et al., 2016; Jing et al., 2017; Santoro et al., 2017).

### Stimulation of MGII in the DCM Layer by Vertical Mixing With Deeper Waters

Phylogenetic analyses have identified the presence of two major groups within MGII, and these groups are referred to as MGIIa and MGIIb (Massana et al., 2000; Martin-Cuadrado et al., 2008; Galand et al., 2009; Belmar et al., 2011). Previous work suggests that the abundances of MGIIa and MGIIb vary seasonally and that these two groups partition niches. For example, Galand et al. (2010) found that MGIIb members developed in nutrient-enriched waters during winter mixing, when phytoplankton blooms occurred. In contrast, MGIIa members were abundant in nutrient-depleted waters during summer stratification when the phytoplankton stocks were relatively low. Furthermore, MGII is relatively abundant in surface waters (DeLong, 2006) and within DCM layers (Martin-Cuadrado et al., 2015; Orsi et al., 2015) in oligotrophic seas. Our findings revealed that active MGIIb members were predominant in the DCM in early November after additional substrates had been brought from 400 m waters.

Although there are no cultured representatives of MGII *Euryarchaeota*, metagenomic sequencing provides a powerful strategy for uncovering the metabolic potential of MGIIa and MGIIb. For example, two draft genomes of thalassoarchaeal fosmid clones from the DCM in the Mediterranean Sea indicated that these taxa have a non-motile photoheterotrophic lifestyle (Martin-Cuadrado et al., 2015). Zhang et al. (2015) summarized the key metabolic functions of MGII, with an emphasis on MGII metabolic genes for the TCA cycle. A relatively high abundance of sequences representing active *Ca.* Nitrosopelagicus brevis strain CN25 was observed in Con-RNA (42.39%) and DCM-RNA (19.42%) samples; this strain actively fixes inorganic carbon via the 3HP/4HB cycle, which leads to the production of acetyl-CoA (Santoro et al., 2010). Thus acetyl-CoA can serve as a precursor for the TCA cycle in MGII members. In our study, five dominant MGI-related OTUs (OTU2, OTU3, OTU8, OTU14, and OTU42) in either MSW-RNA or MSW-DNA samples had 99.3–100% similarity with clones retrieved from the free-living microbial community at a depth of 670 m at a Pacific site (Table 1 and Figure 6). The radiocarbon signatures of thaumarchaeal DNA demonstrated that MGI members derive the majority of their carbon from inorganic carbon fixation from the same site (Hansman et al., 2009). A recent quantitative study of the *amoA* and *accA* genes in the western Pacific revealed similar distributional trends at depths greater than or equal to 100 m as those documented in our study (Zhang et al., 2020). Our sequencing data showed that in 400 m, the distribution trend of 16S rRNA, *amoA*, and *accA* genes was highly consistent, confirming that the ammonia oxidation process of AOA and the fixation of carbon dioxide were mutually coupled in our culture experiments (Supplementary Figure S1). The growth of certain heterotrophic MGII members in DCM-RNA samples could be quickly stimulated by obtaining fresh organic carbon after being mixed with 400 m deep waters. We also found that these five MGI OTUs were almost absent from DCM-RNA and Con-RNA samples, which may explain why the relative abundance of MGII in the DCM-RNA sample was at least threefold greater than that in the controls at 24 h. The DOC in the DCM group was dramatically consumed (Figure 3), indicating that MGII also promoted the degradation of organic matter in the DCM layer after being stimulated. Our results are consistent with previous observations of MGIIb bloomed in the DCM layer after water column mixing during the winter (Martin-Cuadrado et al., 2015). We infer that the marked changes in specific MGII taxa in the DCM-RNA sample occurred in response to vertical mixing.

### Active MGIII Members in the DCM Layer With or Without Vertical Mixing

There were two OTUs represented by more than 1% of the reads from only active archaeal communities in the DCM layer, either with or without vertical mixing (Table 1). Panoceanic OTUs are defined as clones that have identical or nearly identical 16S rRNA genes that have been collected from distant geographical locations (Martin-Cuadrado et al., 2008). OTU9 shared 99.3% similarity with the low-GC genomic clone AD1000-40-D7 collected from a depth of 1000 m in the Adriatic Sea (EU686628). Clone AD1000-40-D7 belonged to the panoceanic OTU D and has a global distribution (Martin-Cuadrado et al., 2008). OTU1 had 99.8% similarity with clone AU1370-28 (JQ181946), which was from surface seawater in the South Pacific Gyre and was the only MGIII clone of the 38 total archaeal clones (Yin et al., 2013).

The first two MGIII clones were retrieved from 500 to 3000 m depths in the Northeastern Pacific in 1997 (Fuhrman et al., 1992). Until Galand et al. (2009, 2010) reported the presence of MGIII *Euryarchaeota* in the photic zone, MGIII members had generally been considered low-abundance members of archaeal communities in deep mesopelagic and bathypelagic waters (Massana et al., 2000; López-García et al., 2001; Martin-Cuadrado et al., 2008). As no representative of MGIII *Euryarchaeota* has been cultivated to date, our knowledge of the metabolic capacity of deep-sea MGIII taxa has been based on comparative metagenomic analyses (Martin-Cuadrado et al., 2008) or constructed partially to nearly completed genomes and transcriptomes (Li et al., 2015). The epipelagic MGIII genomes not only contain numerous photolyase and rhodopsin genes but also harbor enzymes for glycolysis, the carboxylic acid cycle, and the uptake and degradation of peptides and lipids, indicating a photoheterotrophic lifestyle (Haro-Moreno et al., 2017). In our study, MGIII OTUs 1 and 9 had low relative abundances in the total archaeal communities, and incubation with or without vertical mixing resulted in similar shifts in these two representative MGIII OTUs in the DCM layer (Table 1). Thus, if these specific MGIII taxa prefer to utilize substrates provided by other microbes in the DCM layer, vertical mixing might not greatly stimulate the growth of these MGIII members.

### Potential Link Between AOA and MGIIb

The fixation of CO_2_ by marine microbes through chemoautotrophic pathways is an important process for providing fresh organic carbon in the deep sea, and the ammonia oxidation process of AOA may be an important energy source for the fixation of deep-sea organic carbon (Herndl et al., 2005; Reinthaler et al., 2010; Könneke et al., 2014). The *amoA* gene encodes the alpha subunit of a key enzyme in the ammonia oxidation process: AMO (Jung et al., 2014). The *amoA* gene has stronger specificity and higher resolution than the 16S rRNA gene and can more accurately reflect the community structure and distributional characteristics of AOA in environmental samples (Mosier and Francis, 2008; Dang et al., 2010). In the genomes of “*Candidatus* Nitrosopelagicus brevis strain CN25,” “*Candidatus* Nitrosopumilus maritimus,” and “*Candidatus* Cenarchaeum symbiosum,” a new autotrophic carbon fixing mechanism was identified, the 3-hydroxypropionate/4-hydroxybutyrate pathway (Hallam et al., 2006; Walker et al., 2010; Hu et al., 2011b; Santoro et al., 2015). The *accA* genes encoding acetyl-CoA (acetyl-CoA) carboxylase (one of the key enzymes in this pathway) have been used as phylogenetic markers that reflect the ecological function of MGI. qPCR studies have shown that *accA* gene abundance is related to the abundance of *amoA* across the entire ocean water column (Hu et al., 2011a) and also to the abundance of thaumarchaeal 16S rRNA and the CO_2_ fixation rate (Bergauer et al., 2013; Zhang et al., 2020). In our experiment, the parallel niche differentiation of autotrophic archaea and an AOA was confirmed by these genetic markers (*aacA* and *amoA*), showing that the DCM layer and the 400-m layer were dominated by different AOA groups (Figure 4 and Supplementary Figure S1).

The photoautotrophic cyanobacteria (Flombaum et al., 2013) and chemolithoautotrophic AOA (Karner et al., 2001) represent two major groups of marine planktons that are responsible for a considerable fraction of primary production in the global ocean. In our incubation systems, almost no marine cyanobacterial sequences were detected (Supplementary Figure S4), and AOA was the main microbial group making organic matter available for heterotrophic microorganisms. Since the MGII metabolic genes include coding functions related to the TCA cycle, the intermediate acetyl-CoA produced during carbon fixation through the 3HP/4HB pathway can be directly involved in the metabolism of MGII as a precursor (Zhang et al., 2015). The stimulation of MGII growth and DOC degradation were observed in our culture experiment (Figures 3, 5). In contrast to the Control and MWS treatments, two specific MGIIb taxa within the DCM group, OTU90 and OTU73, grew rapidly after receiving fresh substrate from 400 m water (Figure 6); this growth coincided with a rapid decline in DOC in the DCM group compared with the other two groups in the culture experiment (Figure 3). Phylogenetic analysis revealed that OTU90 and 73 are photoheterotrophs of the *Ca.* Thalassarchaeaceae family in the genera O1 and O4, respectively (Rinke et al., 2019). Given the distinct AOA communities in the DCM and 400 m layers, we hypothesize that the AOA in these two layers might release different kinds of organic compounds into surrounding waters and that the vertical mixing process helped pump these substantial substrates from deep water to DCM layers for the growth of certain members of MGIIb in the DCM layer. Recent studies examining the metabolic interactions between marine AOA and heterotrophic bacterial groups support our speculations (Bayer et al., 2019a,b; Reji et al., 2019). Our results highlight the potential role of vertical mixing in linking the uncultured AOA in mesopelagic water and MGIIb in the DCM layer.

### Possible Mechanism of Marine Group II *Euryarchaeota* Stimulation by Mesoscale Eddy-Fueled Organic Carbon

**FIGURE 7:**
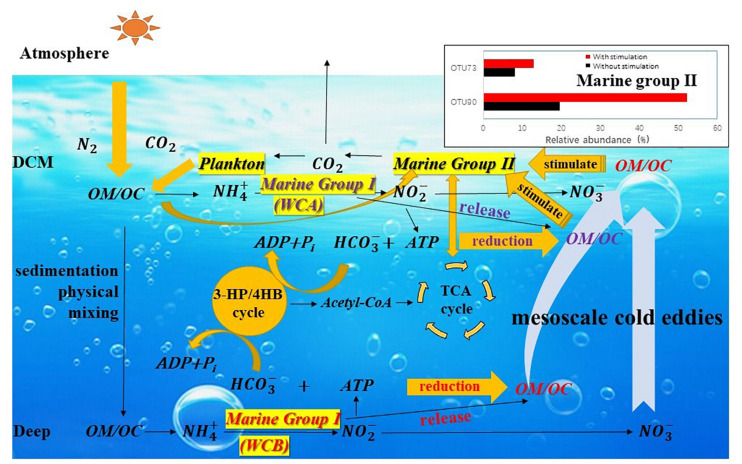
The possible mechanism by which the growth of Marine Group II *Euryarchaeota* is stimulated by carbon flow affected by mesoscale eddies. Particulate organic matter (POM) produced in the upper ocean enters the ocean interior by sedimentation and physical mixing and releases NH_4_^+^ under the action of heterotrophic microorganisms. MGI *Thaumarchaeota* couples the oxidation of ammonia with carbon fixation in HCO_3_^–^ through the 3HP/4HB pathway, and the intermediate acetyl-CoA can act as a precursor of the TCA cycle and participate in the metabolism of MGII. Much organic matter produced by ammonia-oxidizing archaea stored in mesopelagic water is brought into the DCM layer by the mesoscale process. This new organic carbon provides a carbon source for heterotrophic MGII members and rapidly stimulates the growth of specific MGII taxa. The CO_2_ produced during degradation is released into the atmosphere by sea–air exchange or supplied to plankton of the DCM layer.

The particulate organic matter produced in the upper ocean enters the interior of the ocean (below the euphotic layer) by sedimentation and physical mixing (Eppley and Peterson, 1979; Ducklow et al., 2001) and releases NH_4_^+^ under the action of heterotrophic microorganisms (Karl et al., 1984; Kuypers et al., 2018). MGI *Thaumarchaeota* couple the oxidation of ammonia with carbon fixation so that organic matter is synthesized and stored in the interior of the ocean (Figure 7). CO_2_ is fixed in the form of HCO_3_^–^ through the 3HP/4HB pathway, and the intermediate acetyl-CoA can act as a precursor of the TCA cycle and participate in the metabolic processes of MGII (Santoro et al., 2010; Li et al., 2015; Zhang et al., 2015). Much organic matter produced by AOA that is only stored in mesopelagic water is brought into the DCM layer by the mesoscale eddies, and this new organic carbon provides a carbon source for heterotrophic MGII members, rapidly stimulating the growth of MGII members. CO_2_ produced during degradation is released into the atmosphere by sea–air exchange or is supplied to the plankton of the DCM layer (Offre et al., 2013; Zhang et al., 2015). Niche partitioning of either MGI or MGII was observed in this study, and the mesoscale eddies are considered the main mechanism by which organic matter from the interior of the ocean is brought to the DCM layer. Therefore, the simulation of this physical process would facilitate predictions of regional carbon flow under the influence of mesoscale eddies and how the flow might affect biogeochemical processes in regional and global oceans.

## Conclusion

In this study, we demonstrated the distinct niches occupied by autotrophic MGI *Thaumarchaeota* between the DCM layer and mesopelagic water. We have shown that new substrates obtained by the upwelling of seawater by mesoscale cold eddies quickly stimulated the growth of specific MGIIb euryarchaeal taxa at the DCM layer. Given the non-motile lifestyles of MGI in the open ocean (Lehtovirta-Morley et al., 2011) and MGII in the DCM layer (Martin-Cuadrado et al., 2015), we hypothesize that this physical process is the major contributor bringing this new organic matter from the interior of the ocean into the upper layer. Our results provided novel insights into the mechanisms underlying shifts in active archaeal community composition by vertical mixing in the western Pacific Ocean. Additional studies of the enrichment and isolation of representative AOA and MGII as well as metagenomics analysis will strengthen our understanding of how the interaction between these two significant archaeal groups responds to mesoscale eddy perturbations in the open ocean.

## Data Availability Statement

The datasets presented in this study can be found in online repositories. The names of the repository/repositories and accession number(s) can be found in the article /Supplementary Material.

## Author Contributions

JD, QY, YW, MZ, and JZ contributed to research design and sample collection. JD performed the culture experiment. JD and QY analyzed the microbial data. JD, MZ, and YW contributed to the physical and chemical data analyses. JD, QY, YW, and JZ participated in manuscript writing. All authors provided significant input on the final manuscript.

## Conflict of Interest

The authors declare that the research was conducted in the absence of any commercial or financial relationships that could be construed as a potential conflict of interest.

## Footnotes

1 http://drive5.com/uparse/

2 http://rdp.cme.msu.edu/

3 https://blast.ncbi.nlm.nih.gov/Blast.cgi

4 www.i-sanger.com
