# Supplementary material for: Simulation of Enhanced Growth of Marine Group II Euryarchaeota From the Deep Chlorophyll Maximum of the Western Pacific Ocean: Implication for Upwelling Impact on Microbial Functions in the Photic Zone
Source: Front Microbiol. 2020 Sep 11;11:571199. doi: 10.3389/fmicb.2020.571199 (PMC7516215; doi:10.3389/fmicb.2020.571199)
Supplement: Supplementary file 1 [file Table_1.DOCX]

**Table S1.** DCM layer seawater (90 m) and mesopelagic seawater (400 m) were collected by a CTD. Salinity, temperature and oxygen data were obtained from CTD probe detectors. DOC were detected by organic carbon (TOC) analyzer, Nutrients (silicate, phosphate and nitrate) were determined photometrically by an auto-analyzer.

, dissolved organic carbon

|  |  |  |  |  |  |  |  |
| --- | --- | --- | --- | --- | --- | --- | --- |
| **Level** | **Salinity** | **Temperature** | **Oxygen** | **DOC** | **NO_3_^-^** | **PO_4_^3-^** | **SiO_3_^2-^** |
| **(m)** | **(PSU)** | **(**°C**)** | **(mg/L)** | **(μmol/L)** | **(μmol/L)** | **(μmol/L)** | **(μmol/L)** |
| **90** | **34.8** | **24** | **5.2** | **74** | **0.82** | **0.27** | **1.96** |
| **400** | **34.5** | **7.7** | **3.1** | **47** | **31.76** | **2.14** | **26.78** |

**Table S2.** Results of PCR; “＋” represents successful PCR amplification, and “－”

represents failed PCR amplification. Amplicon sequencing of the V4–V5 hypervariable regions of the archaeal 16S rRNA gene and the archaeal 16S rRNA transcripts was performed.

|  | **1** | **2** | **3** |
| --- | --- | --- | --- |
|  | **cDNA** | **DNA** | **DNA** |
| **Con** | **＋** | **＋** | **＋** |
| **DCM** | **＋** | **＋** | **＋** |
| **MSW** | **＋** | **＋** | **＋** |
| **BlK** | **－** | **－** | **－** |

**Table S3.** Diversity and richness estimators for Illumina libraries.

| Sample ID | | observed OTUs | ace | chao | shannon | Coverage |
| --- | --- | --- | --- | --- | --- | --- |
|  |  |  |  |  |  | (%) |
| 16SrRNA （Archaea） | RCon | 33 | 33.00 | 33.00 | 1.84 | 100 |
|  | DCon | 29 | 39.18 | 36.06 | 0.77 | 100 |
|  | RDCM | 44 | 49.24 | 48.20 | 1.56 | 100 |
|  | DDCM | 32 | 46.64 | 41.83 | 0.27 | 100 |
|  | RMSW | 20 | 29.40 | 22.00 | 1.45 | 100 |
|  | DMSW | 50 | 54.25 | 53.60 | 1.86 | 100 |
| 16SrRNA （Bacteria） | Con | 32 | 93.56 | 94.75 | 2.10 | 100 |
|  | DCM | 20 | 91.94 | 92.00 | 2.51 | 100 |
|  | MSW | 50 | 90.31 | 89.88 | 2.82 | 100 |
| *amoA* | Con | 8 | 9.00 | 7.00 | 1.15 | 100 |
|  | DCM | 26 | 19.00 | 19.00 | 1.23 | 100 |
|  | MSW | 53 | 50.00 | 50.00 | 2.95 | 100 |
| *accA* | Con | 19 | 16.00 | 16.00 | 1.06 | 100 |
|  | DCM | 33 | 26.50 | 26.50 | 1.59 | 100 |
|  | MSW | 24 | 22.00 | 22.00 | 1.51 | 100 |

**OTU, operational taxonomic units; Chao, species richness; ACE, abundance-based coverage estimator.** **The R before the sample group name represents RNA (active), and D stands for DNA (total). We use averages in the table.**


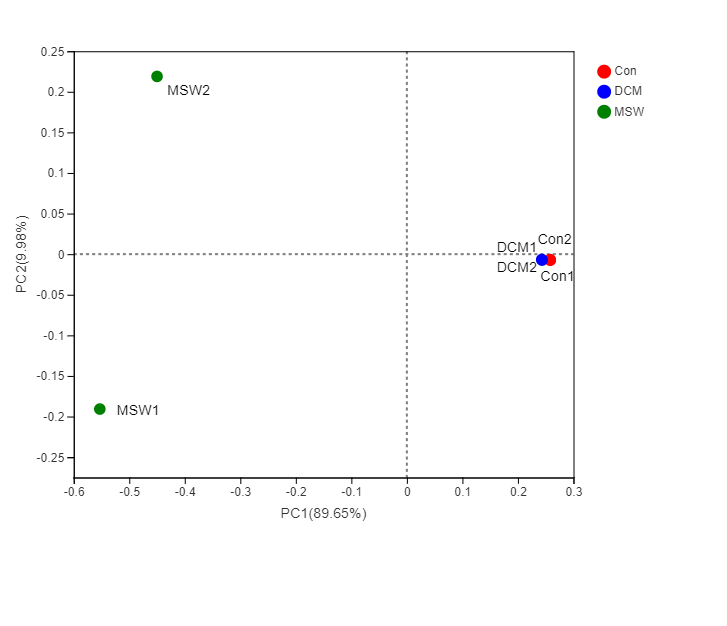
A
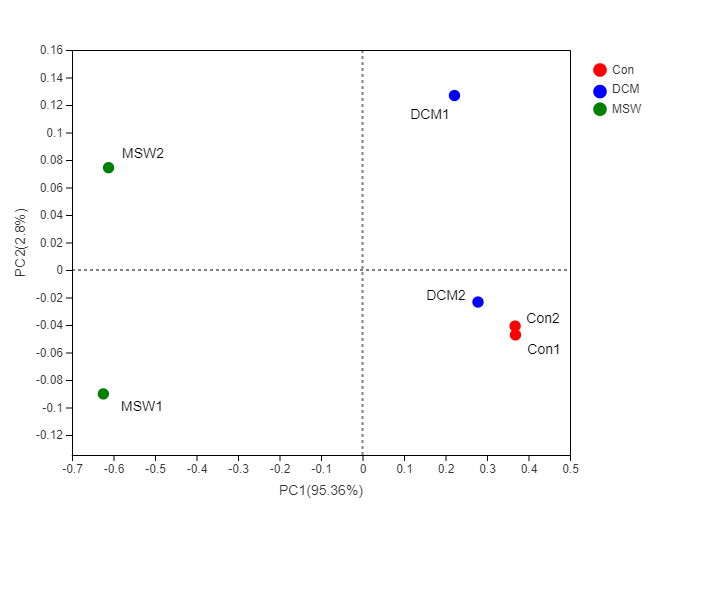
B

**Figure S1.** Principal coordinates analysis (PCoA) based on a Bray-Curtis distance matrix representing differences in the community structure of three groups at the OTU level, A, *amoA*; B, *accA*.


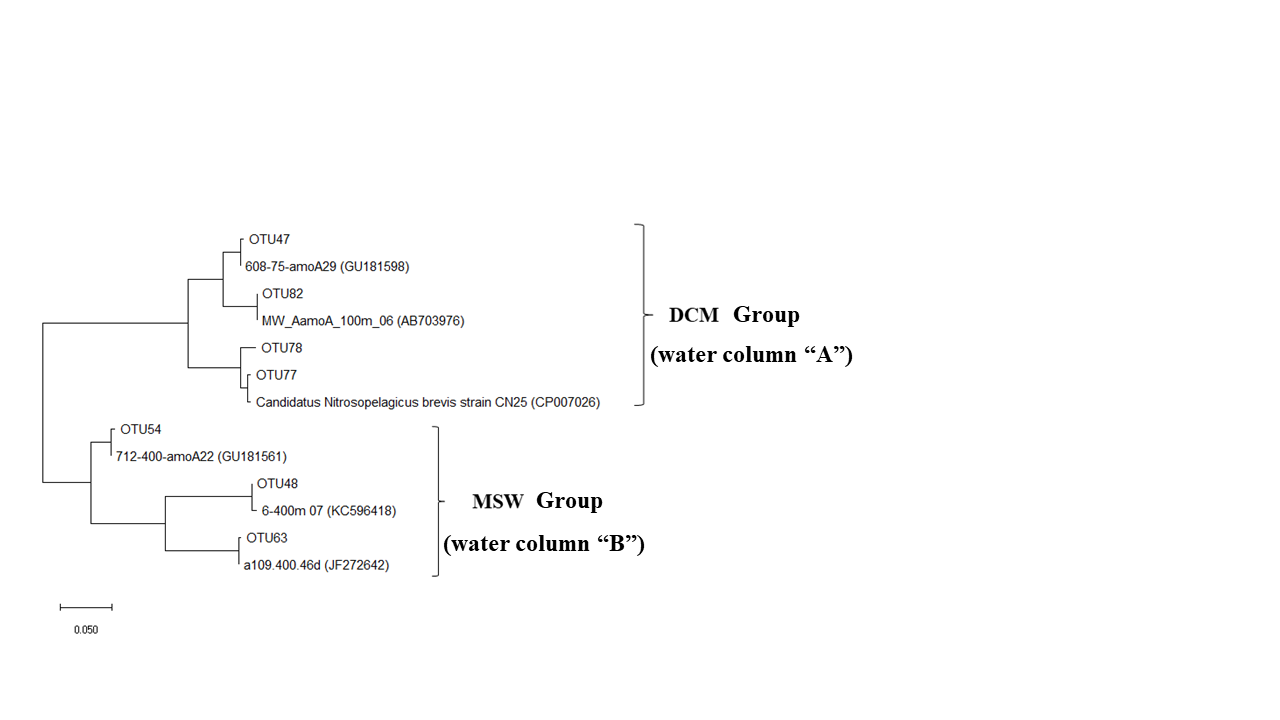


**Figure S2.** Neighbor-joining phylogenetic tree based on AOA *amoA* gene sequences showing the phylogenetic relationship among the representative OTUs and reference AOA *amoA* gene sequences retrieved from NCBI GenBank. The top five OTUs in each group were selected.


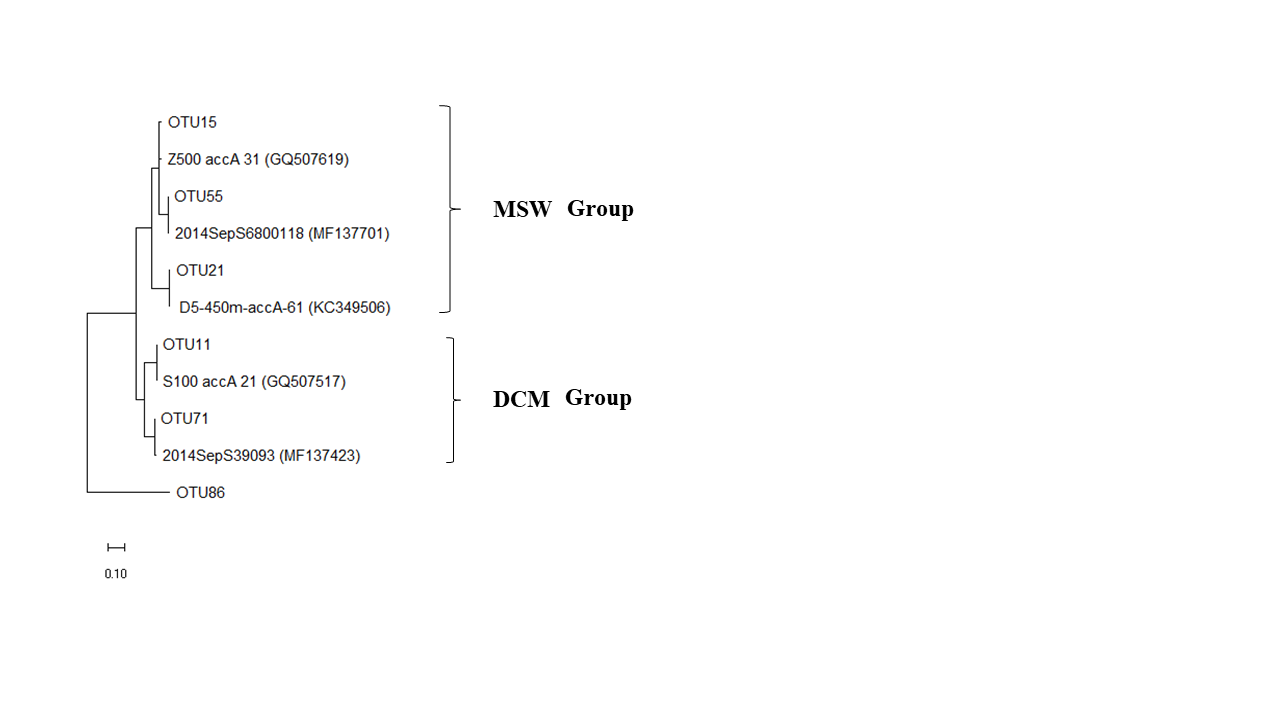


**Figure S3.** Neighbor-joining phylogenetic tree based on *accA* gene sequences showing the phylogenetic relationship among the representative OTUs and reference *accA* gene sequences retrieved from NCBI GenBank. The top three OTUs in each group were selected.


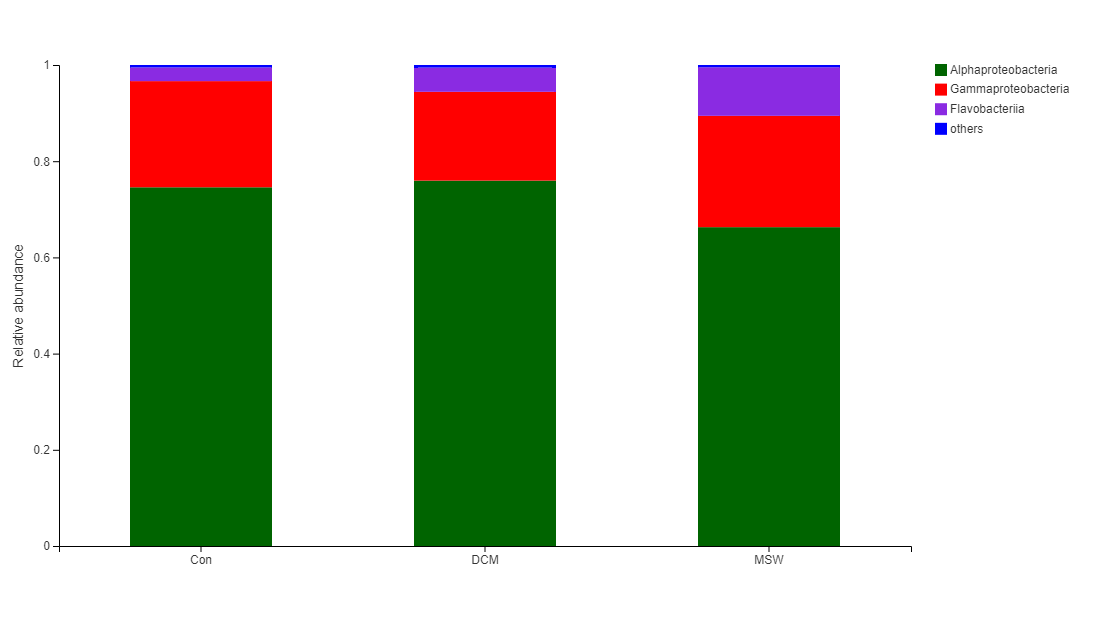


**Figure S4.** Distributions of class-level bacterial taxa of culture experiment. Bars represent the relative abundance of Illumina sequences representative of each class. The proportion of each sequence is the mean value of duplicate analyses.
